# Dynamic survival outcomes of the tall-cell variant of papillary thyroid carcinoma patients after surgery

**DOI:** 10.3389/fendo.2025.1517907

**Published:** 2025-03-24

**Authors:** Yuxiang Xue, Yizhen Zhuang, Shengxiang Chen

**Affiliations:** ^1^ Department of Thyroid and Breast Surgery, The Third Affiliated Hospital of Wenzhou Medical University, Ruian, China; ^2^ Department of Medical Record Office, The Third Affiliated Hospital of Wenzhou Medical University, Ruian, China; ^3^ Department of Gastrointestinal Surgery, The Third Affiliated Hospital of Wenzhou Medical University, Ruian, China

**Keywords:** tall-cell variant, papillary thyroid carcinoma, prognosis, conditional survival, nomogram

## Abstract

**Background:**

Tall cell variant (TCV) represents the predominant aggressive subtype of papillary thyroid carcinoma (PTC). This study aimed to precisely characterize the evolving survival prognosis of these patients using extensive long-term follow-up data from a large cohort.

**Methods:**

Utilizing the Surveillance, Epidemiology, and End Results (SEER) database, a cohort of 1004 eligible TCV patients diagnosed from 2004 to 2016 were included in this investigation. Conditional survival (CS) analysis was used to describe the evolving nature of survival changes for long-term TCV survivors. Following this, the cohort was divided into training and validation sets using a 7:3 ratio. The least absolute shrinkage and selection operator (LASSO) model was utilized to identify prognostically significant factors, which were subsequently integrated to construct a CS-nomogram model. Multiple evaluation methods, including calibration curves, the area under the receiver operating characteristic (ROC) curve, C-index, and decision curve analysis (DCA), were employed to assess the performance of this model.

**Results:**

Among included patients, the Kaplan–Meier method estimated a 10-year OS rate at diagnosis of 85%. In contrast, the CS analysis revealed annual increases, with survival rates improving from 85% at the initial diagnosis to 88%, 90%, 91%, 92%, 94%, 95%, 97%, 99%, and 99% for patients surviving 1 to 9 years after diagnosis, respectively. Through LASSO regression analysis, this study identified age, sex, N status, M status, AJCC stage, tumor size, surgery and radioactive iodine as key predictors for developing the CS-based nomogram. Calibration curves, ROC curves, C-index values, and DCA further determined nomogram model’s effectiveness and reliability. Moreover, based on this CS-nomogram, we calculated risk scores for each patient and used risk scores to categorized patients into high- and low-risk groups in both training and validation cohorts. The Kaplan-Meier analysis with log-rank tests further validated the prognostic discriminative power of our risk stratification.

**Conclusions:**

The findings of our study comprehensively outlined the 10-year CS outcomes for TCV patients, revealing a steady increase in 10-year OS corresponding to each additional year of survival in TCV survivors. We also developed a CS-nomogram model, an individualized tool integrating time-varying covariates and patient-specific characteristics delivers real-time prognostic information tailored to each TCV patient.

## Introduction

Papillary thyroid carcinoma (PTC) is the most prevalent type of endocrine cancer, with a rising occurrence worldwide, positioning it to become the fourth most common cancer globally (1–4). In the past four decades, multiple subtypes of PTC that demonstrate varying tumor behaviors and clinical courses have been discovered. Notably, variants including tall cell variant (TCV), hobnail, columnar, diffuse sclerosing, and solid types have exhibited more aggressive traits (1, 5–7). Among these aggressive variants of PTC, TCV stands out as the predominant subtype with its prevalence ranging from 1% to 19% across studies due to variations in pathological classification systems (8–11). Recent studies indicated an annual increase in the incidence of TCV (9, 12). Furthermore, several researches had indicated that TCV tended to show more aggressive biological behavior and a less favorable prognosis than classical PTC (1, 8); thus, tumors of this type should be classified and studied independently to accurately characterize their features and prognosis.

Currently, many studies present survival rates as fixed, calculated from the time of diagnosis or surgery, and assume that the risk of postoperative mortality or recurrence remains constant (13); however, there is a growing emphasis in recent literatures on the dynamic nature of tumor prognostic risk (14–16). Thus, evaluating prognosis only at baseline for long-term survivors fails to capture the evolving nature of survival changes. Conditional survival (CS) tackles this issue by projecting the likelihood of survival for a certain number of years after diagnosis or treatment, taking into consideration the duration the patient has already survived (17, 18). To our knowledge, no studies have yet explored CS outcomes for patients with TCV. Analyzing CS outcomes for TCV can reveal how prognostic factors change over time. By tailoring survival predictions based on the duration of survival already achieved by the patient, this method offers more personalized and accurate prognostic assessments. Moreover, understanding risk variations at different stages of survival helps in formulating more effective follow-up plans and interventions, ultimately improving overall patient management.

Therefore, this study leveraged the advantages of the Surveillance, Epidemiology, and End Results (SEER) database, which provides multicenter and long-term follow-up data, to investigate the prognosis of patients with this type of tumor. And CS analysis was also employed to elucidate the dynamic changes in survival prognosis. Furthermore, we have developed a CS-integrated nomogram model based on clinical, pathological, and treatment characteristics of patients, specifically tailored for real-time prognosis monitoring in this population. By providing clinicians with patient-specific insights and understanding the dynamic disease evolution, our goal is to equip them with the necessary tools to make informed decisions.

## Methods

### Study population

The SEER database, a comprehensive oncology repository, draws from cancer patient data across diverse geographic regions within the United States, offering a wealth of multicenter clinical and epidemiological information. The data for this study were obtained from the SEER databased, which can be accessed at https://seer.cancer.gov/. Patients eligible for inclusion in the study met the following criteria: (1) diagnosis of TCV-PTC according to the International Classification of Diseases for Oncology, Third Edition (ICD-O-3), histology codes 8344/3; (2) diagnosis date between 2004 and 2016; (3) histologically confirmed; and (4) underwent thyroidectomy. Exclusion criteria included patients with non-primary tumors, incomplete clinical data, or invalid follow-up data. The study gathered the following information for each patient: age, sex, race, tumor size, year of diagnosis, tumor size, American Joint Committee on Cancer (AJCC) stage, surgery type, receipt of radioactive iodine (RAI), marital status, survival time in months, and survival status. Since the SEER database does not provide detailed data on treatment, and RAI therapy is a standard treatment for thyroid cancer, we utilized RAI as a surrogate variable in this study.

### Clinical outcome

The primary outcome of the current study was overall survival (OS) and CS. OS was determined starting from the moment of TCV diagnosis until either death from any cause or the most recent follow-up appointment. CS refers to the probability of survival for a patient, given that they have already survived for a specific period after diagnosis, providing a more dynamic and updated perspective on prognosis over time. The formula for CS is expressed as CS(y|x)=S(y+x)/S(x), where S(x) denotes the Kaplan-Meier survival estimate at x years post-diagnosis. For example, CS(2|3) denotes the probability that a patient who has survived 3 years will survive another 2 years, determined by dividing the 5-year Kaplan-Meier OS estimate by the 3-year OS estimate.

### Annual hazard rate analysis

The Annual Hazard Rate (AHR) represents the instantaneous risk of an event occurring within a one-year interval, conditional on survival up to the start of that interval. We employed the AHR analysis to quantify the changes in annual mortality hazard rates over time.

### Statistical analysis

In this study, categorical variables are represented by numerical values and percentages. And we described the survival prognosis of TCV patients and analyzed the dynamic changes in CS prognosis over time.

For the development and validation of the CS-integrated nomogram model, patients were randomly divided into training and validation cohorts at a 7:3 ratio. This allocation was designed to provide a substantial training cohort for effective model development while maintaining a sufficiently sized validation cohort to ensure accurate performance assessment. To identify significant prognostic factors influencing survival outcomes within the training cohort, we employed least absolute shrinkage and selection operator (LASSO) regression analysis, a robust statistical modeling tool. LASSO was chosen for its unique capability to handle high-dimensional data and perform effective variable selection by shrinking less relevant predictors to zero. This approach not only identifies the most impactful prognostic factors but also mitigates the risk of overfitting, ensuring a more reliable and generalizable model. To capture the changing nature of prognostic factors, we integrated time-dependent covariates. Subsequently, the identified prognostic factors were used to establish a novel CS nomogram via the Cox proportional hazards model.

To assess the performance and accuracy of the CS-nomogram, we utilized comprehensive internal validation techniques such as bootstrapping and cross-validation. These techniques were vital for assessing the nomogram’s calibration and discrimination metrics, such as the C-index value and the area under the receiver operating characteristic (ROC) curve, thereby validating its reliability in predicting survival probabilities over time. Moreover, decision curve analysis (DCA) was used to quantify the net benefit of this nomogram model by considering both the true positives and false positives, providing a more nuanced view of the model’s effectiveness.

Our CS-nomogram assigns values to patients’ clinical characteristics and calculates their individualized survival probabilities at specific time intervals by summing these points cumulatively for each patient. We also used the individual scores calculated by the nomogram to perform risk assessments. By determining the optimal cutoff point for these scores, we classified patients into high-risk and low-risk groups. We then described the distribution of risk scores among patients and further validated the accuracy of this risk stratification system.

All statistical analyses were performed with R software and a two-sided P-value of less than 0.05 was deemed statistically significant.

## Results

### Basic characteristics of the patients

The demographic and clinical characteristics of TCV patients in the entire, training and validation cohorts are summarized in **Table 1**. Our study analyzed data from 1004 TCV patients registered in the SEER database from years ranging 2004 to 2016. Among the included patients, those aged 40-60 years comprised nearly half of the cohort, with women accounting for an overwhelming 72.5% of the population, and the majority being of white ethnicity (84.9%). Regarding tumor characteristics, 45.7% of patients were classified as AJCC stage III/IV, and the majority of tumors were larger than 2 cm, accounting for 53.7%. All these patients underwent surgical treatment, with 92.8% receiving total thyroidectomy. Additionally, 69.4% of the patients received RAI.

**Table 1:** Baseline characteristics of the entire, training and validation sets.

Variables	Entire (N=1004)	Training (N=702)	Validation (N=302)
Age at diagnosis, y				
<40	269 (26.8%)	189 (26.9%)	80 (26.5%)
≥40 and <60	438 (43.6%)	300 (42.7%)	138 (45.7%)
≥60	297 (29.6%)	213 (30.3%)	84 (27.8%)
Sex				
Male	276 (27.5%)	195 (27.8%)	81 (26.8%)
Female	728 (72.5%)	507 (72.2%)	221 (73.2%)
Race				
White	852 (84.9%)	607 (86.5%)	245 (81.1%)
Others	152 (15.1%)	95 (13.5%)	57 (18.9%)
T				
T1/2	432 (43.0%)	295 (42.0%)	137 (45.4%)
T3/4	572 (57.0%)	407 (58.0%)	165 (54.6%)
N				
N0	525 (52.3%)	362 (51.6%)	163 (54.0%)
N1	479 (47.7%)	340 (48.4%)	139 (46.0%)
M				
M0	976 (97.2%)	682 (97.2%)	294 (97.4%)
M1	28 (2.8%)	20 (2.8%)	8 (2.6%)
AJCC stage				
I	493 (49.1%)	347 (49.4%)	146 (48.3%)
II	52 (5.2%)	32 (4.6%)	20 (6.6%)
III	253 (25.2%)	181 (25.8%)	72 (23.8%)
IV	206 (20.5%)	142 (20.2%)	64 (21.2%)
Tumor size, mm				
<20	465 (46.3%)	324 (46.2%)	141 (46.7%)
≥20 and <40	351 (35.0%)	250 (35.6%)	101 (33.4%)
≥40	188 (18.7%)	128 (18.2%)	60 (19.9%)
Surgery				
STNTT	72 (7.2%)	49 (7.0%)	23 (7.6%)
TT	932 (92.8%)	653 (93.0%)	279 (92.4%)
RAI				
No	307 (30.6%)	214 (30.5%)	93 (30.8%)
Yes	697 (69.4%)	488 (69.5%)	209 (69.2%)
Marital status				
Single	375 (37.4%)	265 (37.7%)	110 (36.4%)
Married	629 (62.6%)	437 (62.3%)	192 (63.6%)

RAI, radioactive iodine; STNTT, Subtotal or Near-total Thyroidectomy; TT, Total Thyroidectomy.

### CS analysis and AHR analysis

**Figure 1:**
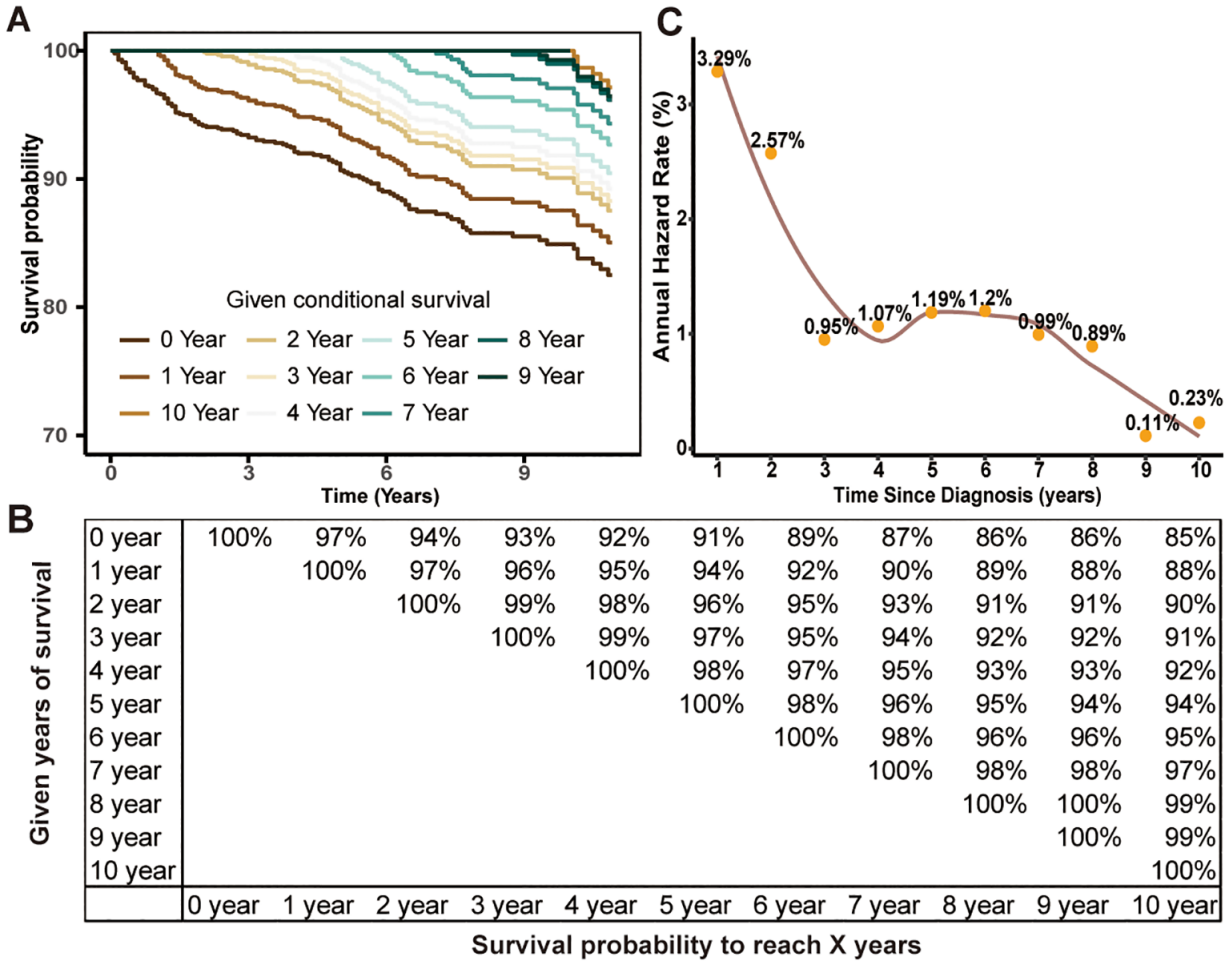
Estimating conditional survival (CS) outcomes in tall cell variant of papillary thyroid carcinoma patients using the Kaplan-Meier method. Conditional survival curves **(A)** adjusted for duration of survival and updated survival data **(B)**. Annual hazard rate change over time **(C)**.

**Figure 2:**
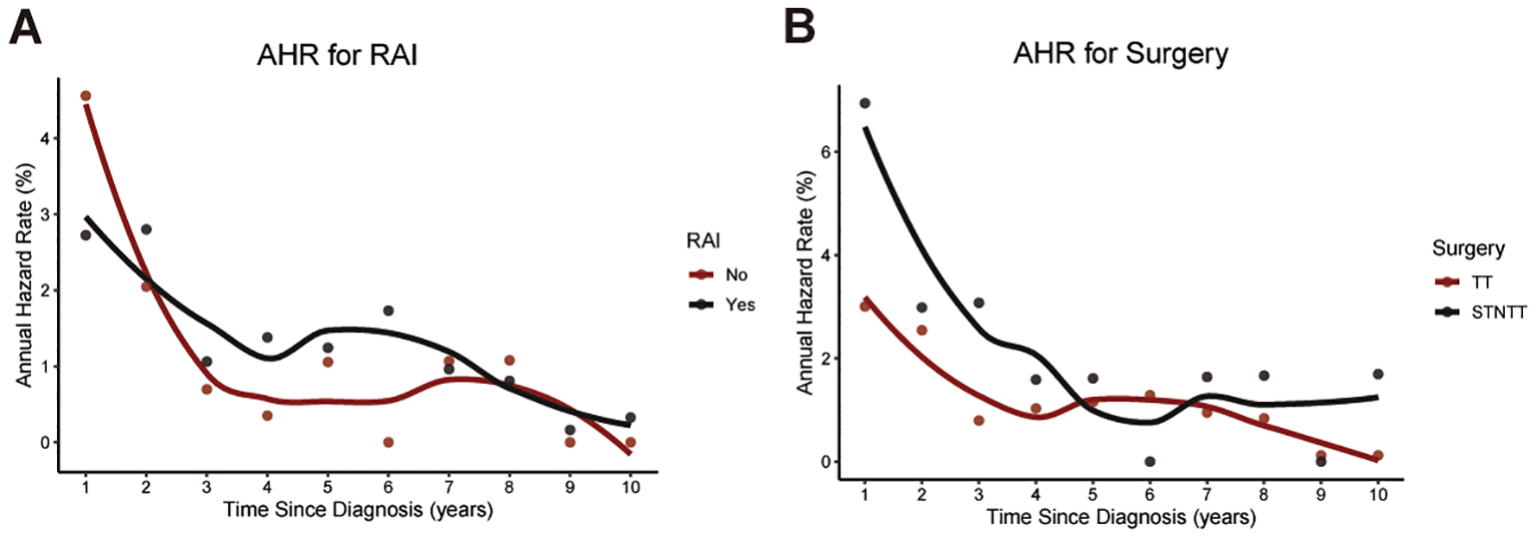
Temporal changes in annual hazard rates associated with RAI therapy **(A)** and surgical intervention **(B)**. RAI, radioactive iodine; STNTT, Subtotal or Neartotal Thyroidectomy; TT, Total Thyroidectomy.

Then, we conducted CS analysis to assess the prognosis of TCV patients. **Figures 1A, B** demonstrated a steady enhancement in real-time survival for TCV survivors over a 10-year follow-up period. The curves in the plot illustrated survival rates adjusted according to their duration of survival. The Kaplan–Meier method estimated a 10-year OS rate at diagnosis of 85%. In contrast, the CS analysis revealed annual increases, with survival rates improving from 85% at the initial diagnosis to CS(9|1)=88%, CS(8|2)=90%, CS(7|3)=91%, CS(6|4)=92%, CS(5|5)=94%, CS(4|6)=95%, CS(3|7)=97%, CS(2|8)=99%, and CS(1|9)=99% for patients surviving 1 to 9 years after diagnosis, respectively. The AHR analysis demonstrated that patients in this cohort experienced a relatively elevated mortality risk during the initial two years post-diagnosis, followed by a gradual decline that eventually stabilized over time (**Figure 1C**). And RAI therapy and total thyroidectomy primarily reduced early mortality rates compared to the untreated group, with a more pronounced effect observed in the initial phases following treatment (**Figures 2A, B**).

### CS-nomogram development and validation

**Figure 3:**
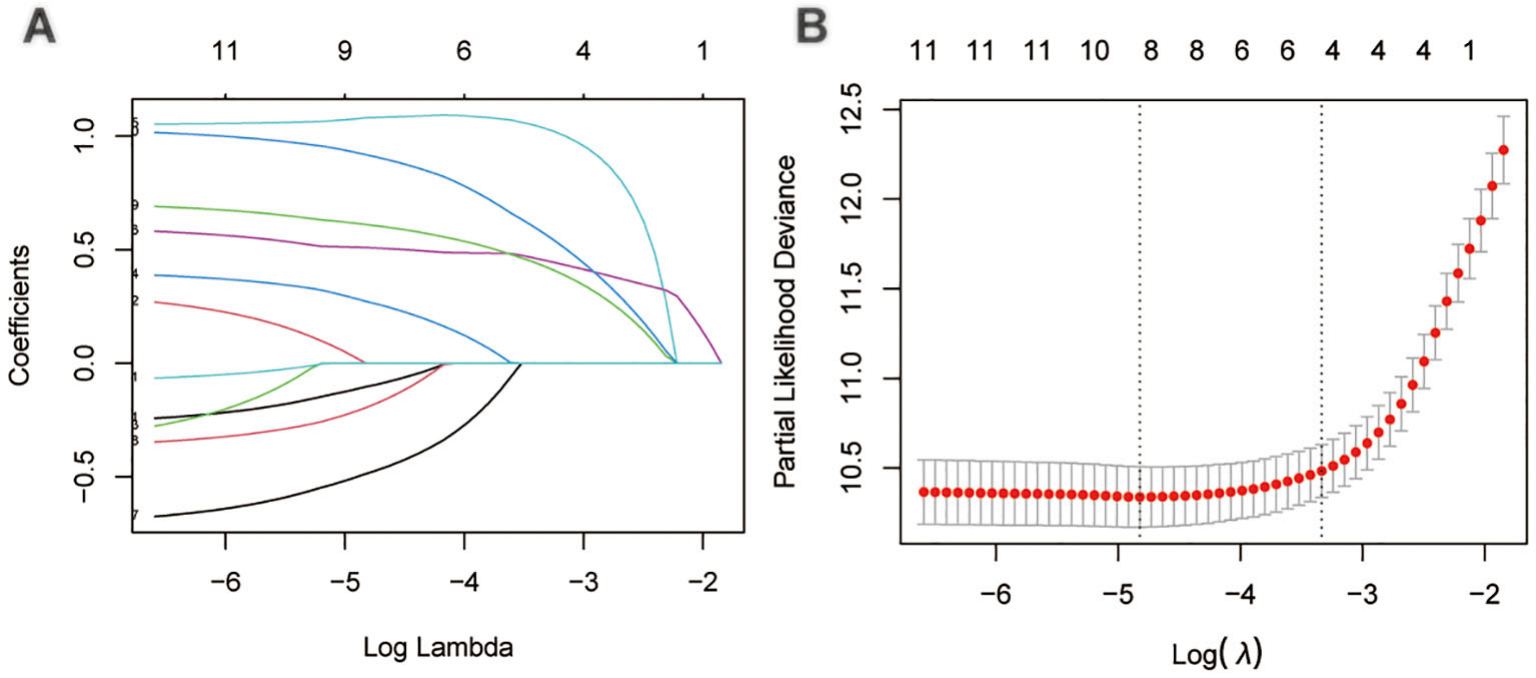
The least absolute shrinkage and selection operator (LASSO) regression analysis for prognostic factors screening **(A, B)**.

**Figure 4:**
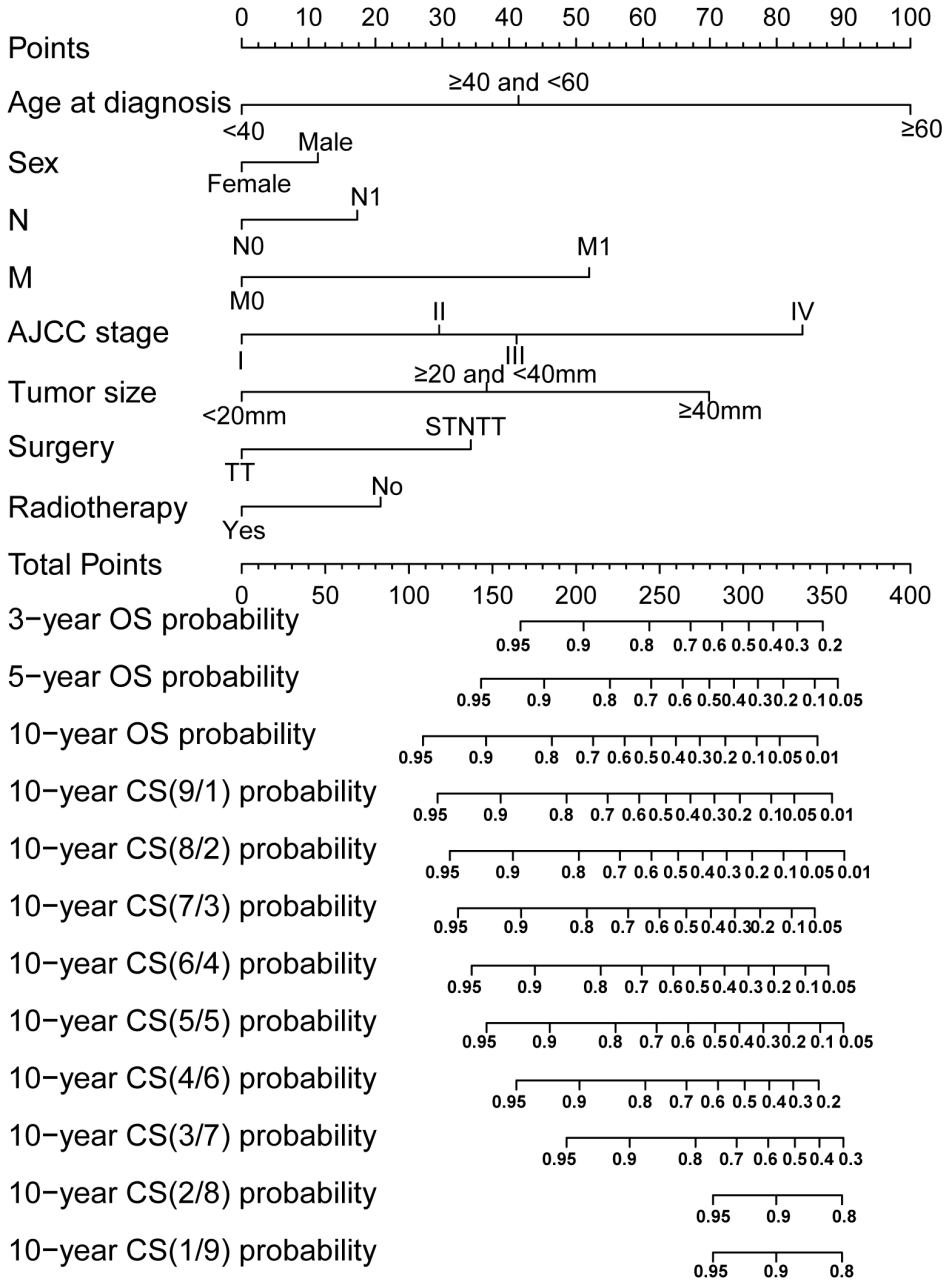
Conditional survival (CS) nomogram for predicting 3-, 5- and 10-year overall survival and 10-year CS for TCV patients. To use the nomogram, clinicians should first locate the patient’s value for each prognostic factor on the corresponding axis and draw a vertical line to the points axis to assign a score. After summing the scores for all variables, plot the total score on the total points axis and draw a vertical line downward to the outcome axis to obtain the predicted probability. STNTT, Subtotal or Near-total Thyroidectomy; TT, Total Thyroidectomy.

Through LASSO regression analysis, the study identified age, sex, N status, M status, AJCC stage, tumor size, surgery and RAI as key predictors for developing the CS prediction model (**Figures 3A, B**). Subsequent multivariate Cox regression analysis demonstrated the impact of these predictors on survival and was employed to construct the CS-nomogram model (**Table 2**). The results further demonstrated that advanced age, N1 status, M1 status, higher AJCC stage, larger tumor size, subtotal thyroidectomy, and the absence of RAI therapy were significantly associated with poor prognosis, although N stage and RAI did not reach statistical significance in the multivariate regression analysis. Our CS-nomogram visually illustrated how different variables impact conditional survival outcomes and calculated corresponding survival probabilities based on these variables for TCV patients (**Figure 4**).

**Table 2:** Multivariable Cox proportional hazards analysis of risk factors.

Variables	Multivariate analysis		
	HR	95%CI	P
Age at diagnosis, y				
<40	1		
≥40 and <60	2.121	0.493-9.119	0.312
≥60	6.446	1.473-28.207	0.013
Sex				
Male	1		
Female	0.765	0.490-1.196	0.24
Race				
White	1		
Others	1.335	0.687-2.593	0.394
T				
T1/2	1		
T3/4	0.72	0.331-1.563	0.406
N				
N0	1		
N1	1.424	0.836-2.426	0.193
M				
M0			
M1	2.729	1.486-5.011	0.001
AJCC stage				
I	1		
II	1.641	0.376-7.157	0.51
III	2.721	0.887-8.347	0.08
IV	5.988	1.846-19.420	0.003
Tumor size, mm				
<20	1		
≥20 and <40	2.075	1.049- 4.106	0.036
≥40	4.083	2.036- 8.188	<0.001
Surgery				
STNTT	1		
TT	0.49	0.265-0.906	0.023
RAI				
No			
Yes	0.685	0.423-1.111	0.125
Marital status				
Single	1		
Married	0.937	0.603-1.455	0.772

RAI, radioactive iodine; STNTT, Subtotal or Near-total Thyroidectomy; TT, Total Thyroidectomy.

**Figure 5:**
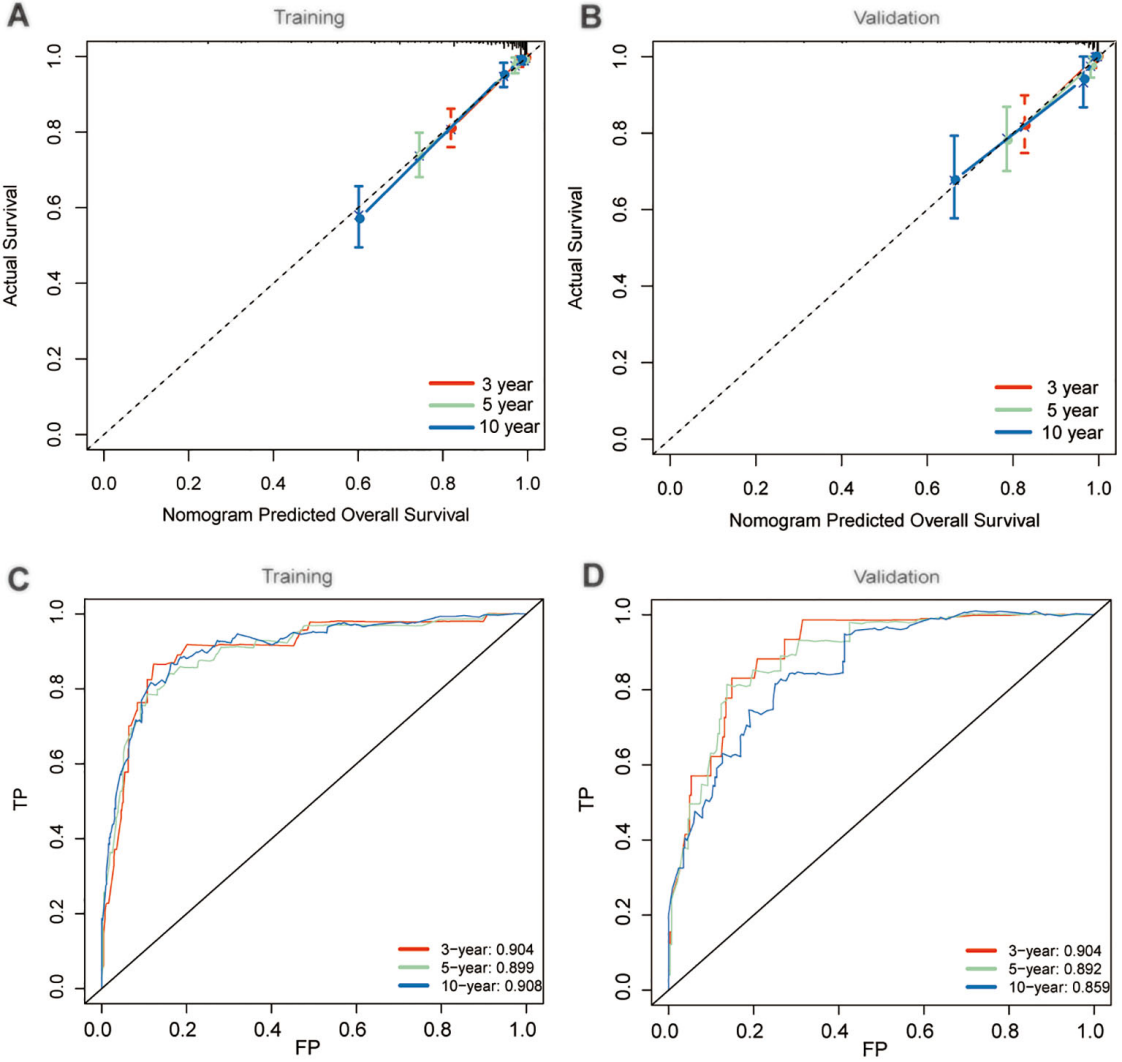
Performance evaluation of nomogram model. Calibration plots in training **(A)** and validation **(B)** cohorts; Time-dependent receiver operating characteristic (ROC) curves in training **(C)** and validation **(D)** cohorts.

**Figure 6:**
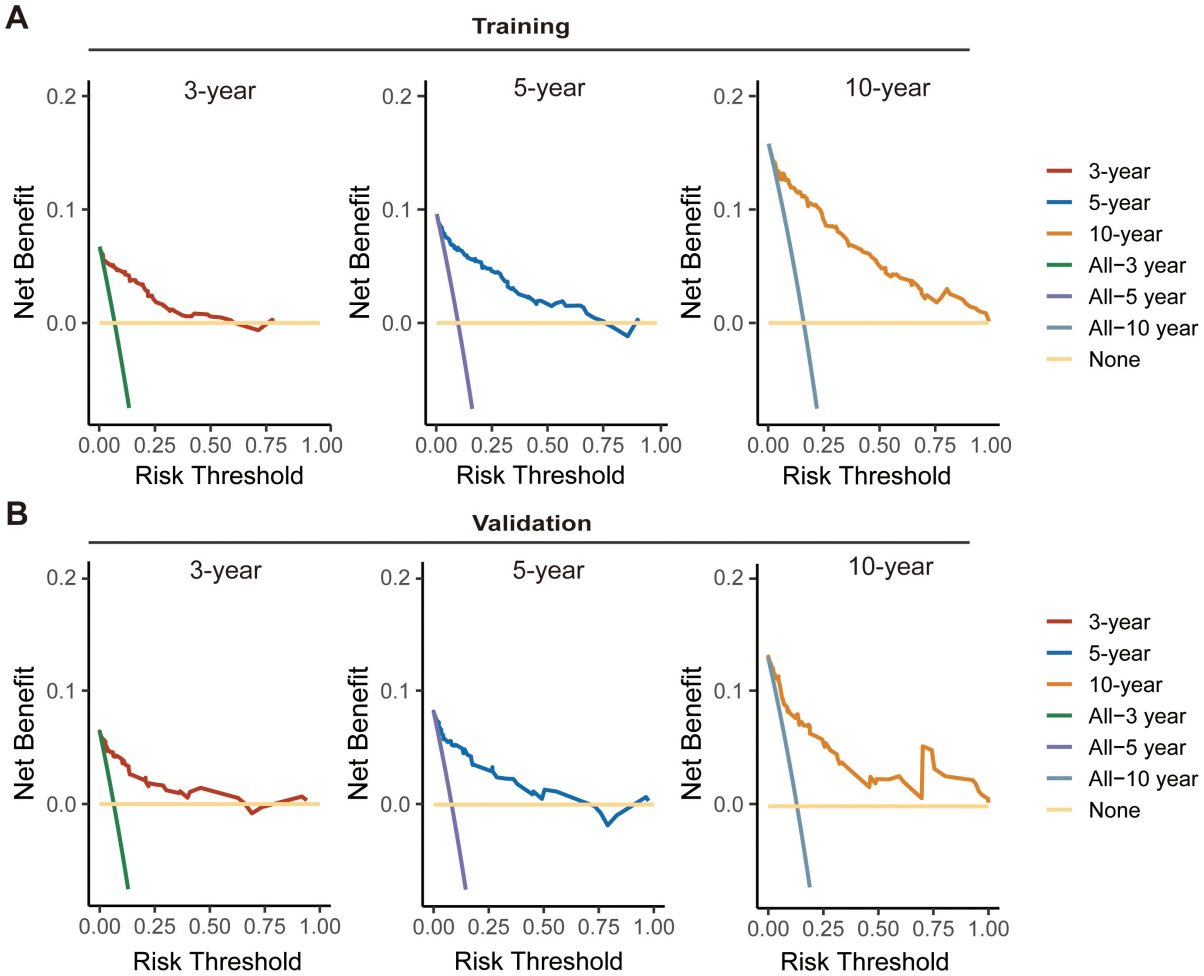
Decision curve analysis (DCA) curves for assessing the clinical usefulness of the nomogram model in training **(A)** and validation **(B)** cohorts.

Considering the consistency and discriminative capability of the nomogram model comprehensively can help determine its effectiveness and reliability. Consistency ensures the stability and credibility of the data and methods used, while discriminative capability ensures the model effectively illustrates the outcome differences among different patients. In our study, the calibration curves demonstrated satisfactory agreement, closely aligning with the ideal 45° line in both training and validation cohorts (**Figures 5A, B**). And C-index value of the model in training cohort was 0.877, and in validation was 0.890, displaying a favorable performance. Meanwhile, time-dependent ROC analysis showed that the 3-year, 5-year, and 10-year AUC values were 0.904, 0.899, and 0.908 in training cohort, and 0.904, 0.892, and 0.859 in validation cohort, respectively (**Figures 5C, D**). DCA further demonstrated that the nomogram yielded greater net benefit in predicting CS outcomes across both the training and validation cohorts (**Figures 6A, B**).

### CS-nomogram-based risk stratification

**Figure 7:**
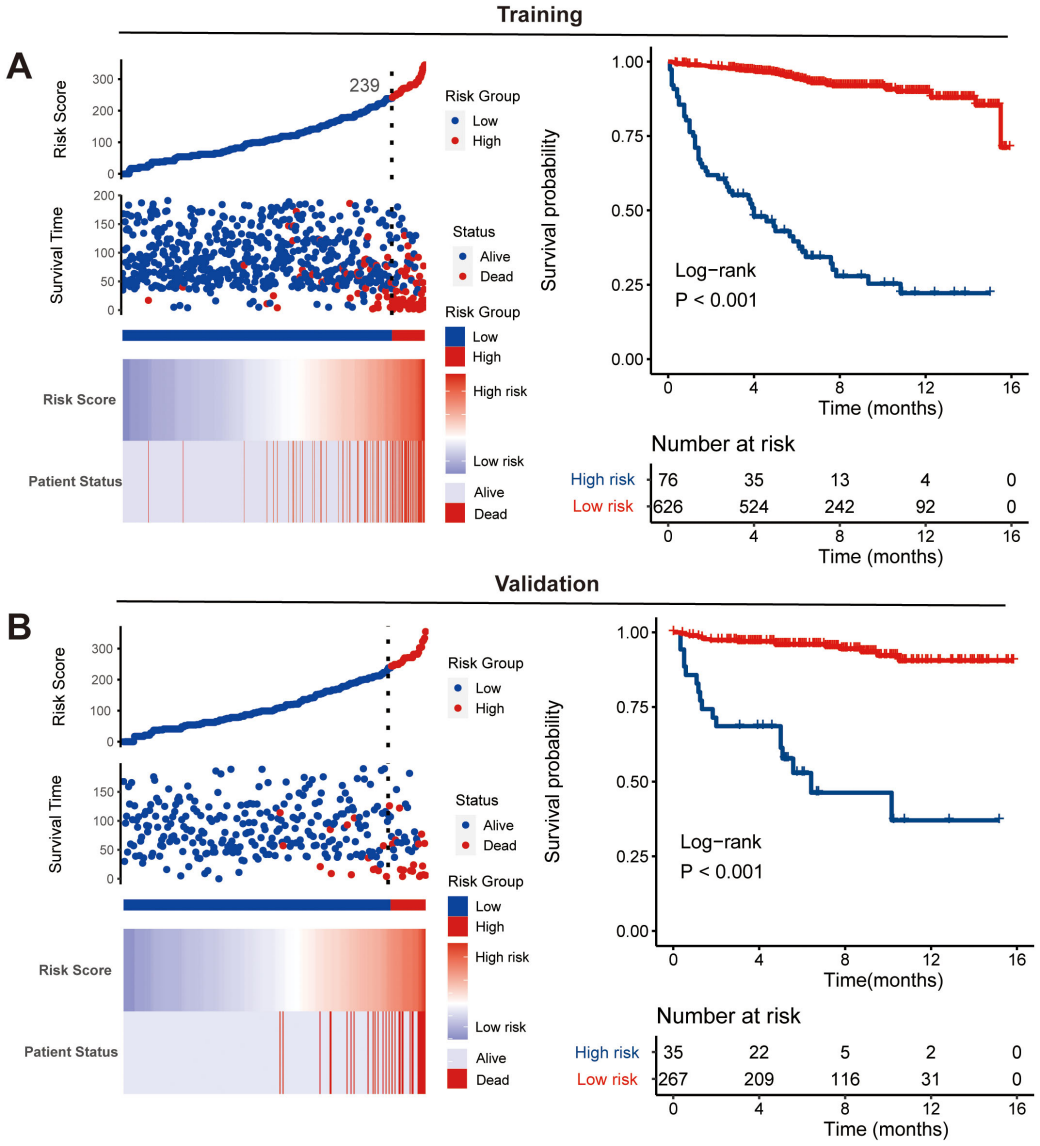
Nomogram-based risk stratification. Risk score distribution and Kaplan-Meier analysis of risk groups along with log-rank tests in training **(A)** and validation **(B)** cohorts.

Using the nomogram model integrated with CS, we calculated risk scores for each patient. By identifying the optimal cutoff point of 239 from risk scores in the training cohort, patients were categorized into high-risk and low-risk groups. This optimal cutoff score also effectively identified high-risk patients in the validation cohort. The distribution of risk patients in both groups is illustrated in **Figures 6A, B**. Additionally, Kaplan-Meier analysis with log-rank tests further validated the prognostic discriminative power of our risk stratification (**Figures 7A, B**).

## Discussion

TCV represents the predominant invasive variation of PTC. However, its rarity and challenges in differentiation from other variants have resulted in most existing literature being comprised of case reports or small case series with diverse diagnostic criteria across individual institutions, thereby restricting our comprehension of TCV (8). Considering the heterogeneity of these different pathological types of PTC, this study separately delineated the TCV cohort and conducted CS analysis, thereby providing more accurate and reliable dynamic prognostic information for these patients. Furthermore, to our knowledge, this study was the first to employ CS analysis to describe the prognosis of this patient group and establish a corresponding predictive model, aiming to provide deeper insights into this cohort. Simultaneously, the SEER database provided a multicenter, large-sample clinical cohort of TCV with extensive follow-up data, greatly enhancing the credibility and applicability of our study results.

In traditional survival analysis, survival estimates were determined based on the tumor status at initial diagnosis, resulting in survival data that remained unchanged during follow-up. However, in reality, the impact of clinical and pathological characteristics on survival prognosis is dynamically changing, leading to changes in patients’ survival probabilities as their survival time progresses (19, 20). Recent studies have indicated that long-term tumor survivors were often more concerned about their real-time survival probabilities (21). Clinicians can optimize clinical management strategies by updating expected survival probabilities based on the patient’s accrued survival time. To ensure an adequate follow-up duration, we selected patients diagnosed between 2004 and 2016 for CS analysis, aiming to provide more precise data. Our study successfully described the 10-year CS outcomes of TCV patients, and we found that there was a consistent enhancement in 10-year OS with each additional year of survival among TCV survivors in this study. Furthermore, our AHR analysis identified that the period of highest mortality risk in TCV patients occurs early after diagnosis (1-2 years), underscoring the critical importance of prompt intervention and rigorous follow-up during this initial phase. Additional treatment-related AHR analysis revealed that both surgical treatment and RAI therapy significantly reduce early mortality rates, highlighting the necessity of timely therapeutic measures to improve patient outcomes. This enables clinicians and patients to comprehend how survival expectations evolve over time, fostering deeper insights into disease progression and the effectiveness of treatments.

Of course, relying solely on CS analysis is inadequate to consider individual differences. We should fully consider the influence of individual clinical characteristics on patient prognosis and integrate them with CS to offer more personalized conditional survival data. A nomogram model can address this by providing a visual representation of how clinical characteristics directly impact survival outcomes (22). Therefore, our study successfully developed a nomogram integrated with CS, providing a more convenient and intuitive tool for clinical management of these patients. The CS-nomogram developed in this study incorporates factors significantly associated with OS in TCV cases. It highlights that age is the most influential variable affecting prognosis, followed by AJCC stage, tumor size, and other factors. This finding are consistent with results reported in previous research (8, 10). Moreover, in clinical practice, a nomogram serves as a practical and user-friendly tool to predict individual patient outcomes based on specific prognostic factors. To use the nomogram, clinicians first locate the patient’s value for each prognostic factor on the corresponding axis and draw a vertical line to the points axis to assign a score. After summing the scores for all variables, the total score is plotted on the total points axis, and a vertical line is drawn downward to the outcome axis to obtain the predicted probability. This visual and quantitative approach allows clinicians to make informed decisions, tailor treatment plans, and provide patients with personalized prognostic information.

For treatment, it is evident that patients undergoing total thyroidectomy indeed show better prognostic outcomes compared to those undergoing subtotal or near-total thyroidectomy. Meanwhile, we also found that RAI had a positive impact on prognosis for these patients in our model. Currently, RAI can enhance the prognosis for some PTC patients, but its effect on the prognosis of TCV patients remains a subject of debate (3, 8). Recently, Holoubek et al. analyzed data from the NCDB on 3,739 patients with TCV and found that patients with larger TCV (>2 cm) derived a survival benefit from RAI, whereas those with smaller TCV did not (23). In contrast, Dai et al. conducted a retrospective analysis using clinical data and propensity score matching (PSM) to balance baseline characteristics between the RAI and non-RAI groups (8). They found that RAI may not improve cancer-specific survival in TCV patients after total thyroidectomy. Also, some researchers suggested that, given the aggressive features often exhibited by TCV patients—such as advanced age, extrathyroidal invasion, and lymph node metastasis—it is advisable for them to undergo total thyroidectomy followed by postoperative RAI treatment. Therefore, future research is needed to further confirm the role of RAI in the treatment of TCV. Our CS analysis tool will aid in adjusting the survival baselines of enrolled patients, making the implementation of randomized controlled trials (RCTs) more scientific and reliable.

Our study has several limitations. First, while the SEER database provides a robust and extensive dataset, it lacks certain critical clinical details, such as data on disease recurrence, tumor molecular biology, detailed tumor characteristics and patient-reported quality-of-life metrics. The absence of these variables may limit the depth of our analysis and hinder a more comprehensive understanding of specific research questions. Additionally, the inability to perform pathological re-evaluation, such as confirming histological subtypes or the proportion of tall cells in TCV, may introduce potential bias. Second, one of the limitations of this study is the lack of detailed information regarding the specific treatment regimens. While SEER provides data on the general use of therapies such as surgery, radiation, and chemotherapy, it does not include detailed information on the treatment protocols. Another limitation is that the SEER database does not provide detailed information on the specific type radiation therapy used, nor does it include data on the number of nodes excised or the specific types of nodal dissection, all of which may impact patient outcomes in different ways. Thirdly, as the data is derived from the United States, the lack of inclusion of individuals from Eastern populations may reduce its generalizability. Lastly, due to the lack of external validation, our analyses may be subject to survival bias. And future international multicenter studies are necessary to further validate our findings.

## Conclusions

Using long-term follow-up data from the SEER database, our study comprehensively analyzed 10-year CS in TCV of PTC, demonstrating a steady improvement in 10-year CS with each additional year of survival. And AHR analysis emphasized the importance of early intervention and tailored follow-up strategies, particularly during the high-risk period within the first 1-2 years post-diagnosis. We also developed a CS-based nomogram, integrating time-varying covariates and patient-specific characteristics, to provide real-time, individualized prognostic predictions. This tool enhances risk stratification and supports personalized treatment planning.

## Data Availability

Publicly available datasets were analyzed in this study. This data can be found here: https://seer.cancer.gov/data-software/.
